# Antibacterial Chitosan Nanofiber Thin Films with Bacitracin Zinc Salt

**DOI:** 10.3390/polym13071104

**Published:** 2021-03-30

**Authors:** Kazutaka Kumamoto, Toshinari Maeda, Satoshi Hayakawa, Nurul Asyifah Binti Mustapha, Meng-Jiy Wang, Yuki Shirosaki

**Affiliations:** 1 Faculty of Engineering, Kyushu Institute of Technology, 1-1 Sensui-cho, Tobata-ku, Kitakyushu, Fukuoka 804-8550, Japan; p109025k@mail.kyutech.jp; 2 Graduate School of Life Science and Systems Engineering, Kyushu Institute of Technology, 2-4 Hibikino, Wakamatsu-ku, Kitakyushu, Fukuoka 808-0196, Japan; toshi.maeda@life.kyutech.ac.jp (T.M.); syifah86@yahoo.com (N.A.B.M.); 3 Graduate School of Interdisciplinary Science and Engineering in Health Systems, Okayama University, 3-1-1 Tsushima-naka, Kita-ku, Okayama 700-8530, Japan; satoshi@okayama-u.ac.jp; 4 Department of Chemical Engineering, National Taiwan University of Science and Technology, No. 43, Sec. 4, Keelung Rd., Taipei 106, Taiwan; mjwang@mail.ntust.edu.tw

**Keywords:** chitosan nanofiber, thin film, antibacterial properties, fibroblast viability

## Abstract

Chitosan nanofiber has a highly uniform structure of 20–50 nm in diameter and shows high dispersibility in water due to its submicron size and high surface-to-volume ratio. The stacked nanofibers film is useful for breathability because it has a gap with a size of several tens of nm or more. However, the chemical bonds between the nanofibers cannot be broken during use. In this study, the thin films were obtained by filtration of chitosan nanofibers and 3-glycidoxypropyltrimethoxysilane (GPTMS) mixture. The addition of GPTMS changed the wettability, mechanical property and stability in water of the thin films. Bacitracin zinc salt (BZ) has been used for the localized dermatological medicines and loaded in the films. BZ interacted electrostatically with the thin films matrix and the release of BZ was controlled by the amount of GPTMS. A higher released amount of BZ showed higher antibacterial effects toward *S. aureus*. The film was also tested their toxicity by L929 fibroblasts. The release of less than 11.9 μg of BZ showed antibacterial effects, but were not toxic for fibroblast cells.

## 1. Introduction

*Staphylococcus aureus* (*S. aureus*, a Gram-positive bacteria) is a major bacterial human pathogen related to skin infections [1,2,3]. Skin and soft tissue infections such as impetigo, folliculitis and cellulitis are developed when *S. aureus* invades the skin following scratches, puncture wounds and burns. Most skin infections are not life-threatening but can cause psychological stress. Early-stage treatments include incising and draining the lesion and treating with antimicrobial drugs, such as oral formulations or ointments; however, side effects such as vomiting and diarrhea occur in some cases. Recently developed antibiotics do not generally cause these serious side effects, but they should generally not be taken with other medicines. In particular, patients taking diabetes and immunosuppressive medicines often experience serious side effects [4,5,6]. Moreover, complete healing can be slow, more than a month in some cases. Even minor side effects can therefore cause distress over these long periods. In addition, in the case of severe vulgaris, the condition may not be completely cured and may remain as a mark accompanied by ridges, depressions and pigmentation. Patients may also leave treatments unfinished because of the long-term nature of the battle with bacteria. For these reasons, it is critical to prevent *S. aureus*-derived skin infections in a facile manner using simple materials, to maintain a high quality of life for patients.

The aim of this study is to prepare an antibacterial thin films that can prevent infection by causative bacteria from the outside environment. The thin film requires the following properties: (1) gas and water vapor permeability for skin respiration, (2) stability and flexibility in air and water environments and (3) inhibition of causative bacteria. To obtain these characteristics, we focused on the combination of chitosan nanofibers and silane coupling agent.

Chitin/chitosan nanofibers, which have marked potential for medical applications because of their characteristic morphology and high specific surface area, were prepared using several methods [7,8]. Chitosan nanofibers prepared using the Star Burst System developed by Sugino Machine Co., Ltd. (Toyama, Japan), are homogeneous in size and have a small diameter, good crystallinity and high purity [9,10]. Chitosan nanofibers are aggregates of microfibers with a width of 3–4 nm—which are bundles of dozens of macromolecules—and form gaps of several tens of nanometers as a result of entanglement. These gaps are expected to be effective for gas or water vapor permeation. However, the hydrogen bonding between chitosan nanofibers is not strong enough to maintain their shape and mechanical properties in wet conditions. Crosslinking is therefore important for the stability of chitosan-based materials. Aldehydes are commonly used to crosslink chitosan [11,12,13]; however, they are highly cytotoxic when used in excess. Shirosaki et al. [14,15,16,17], crosslinked chitosan with 3-glycidoxypropyltrimethoxysilane (GPTMS), a silane coupling agent with epoxy groups, reacts amino groups of the chitosan chains. Silanol (–Si–OH) groups formed by the hydrolysis of the methoxy groups also interact with the amino groups electrostatically. Both interactions are thought to improve the mechanical strength and stability. In addition, the toxicity of GPTMS monomer was found to be 20 times lower than that of glutaraldehyde [17].

Chitosan is commonly known to have antibacterial properties against several bacteria. This behavior is only observed below pH 6.0 because the protonated amino groups interact with microbial cell membranes [18,19]. However, acidic chitosan films have unstable structures under wet conditions and are easily degraded. The pH of skin depends on its condition [20]. The healthy skin surface is pH 4.1–5.8, while infected surfaces can have pH greater than 6.0 because the pathogenic bacteria favor alkaline pH. Therefore, neutral chitosan does not have an antibacterial effect on the infected skin and chitosan films mast be combined with antibacterial drugs to kill the bacteria at neutral pH.

In this study, chitosan nanofibers are crosslinked with GPTMS to prepare the thin films and bacitracin zinc salt (BZ) is introduced into the films as an antibacterial drug. BZ prevents *S. aureus* from growing by suppressing cell wall synthesis and protein synthesis [21]. BZ is expected to interact with the chitosan-GPTMS thin films through electrostatic interactions and hydrogen bonds.

## 2. Materials and Methods

### 2.1. Preparation of the Thin Films

Chitosan nanofiber (BiNFi-s Chitosan, 2 mass% chitosan nanofiber, 20–50 nm average diameter, 80 m^2^·g^−1^ specific surface area) was supplied by Sugino Machine Ltd. (Toyama, Japan). The chitosan nanofiber dispersion was diluted with distilled water to a concentration of 0.2 mass%. An appropriate amount of (3-glycidoxypropyl)trimethoxysilane (GPTMS, 97%, Alfa Aesar, Ward Hill, MA, USA) was hydrolyzed by adding to a 0.25 M acetic acid aqueous solution. Then, various amount of bacitracin zinc salt (BZ, Sigma-Aldrich, St. Louis, MO, USA) were dissolved in the GPTMS/AcOH solution. The obtained solutions and chitosan nanofiber solution were mixed at room temperature for 3 h. The final solutions were filtered through a cellulose nitrate filter membrane (0.20 µm pore size) under vacuum and dried at 40 °C for 1 day to obtain the thin films. The sample compositions and identifying codes are described in Table 1.

### 2.2. Thickness and Surface Morphology of Thin Films

Film thickness (n = 3) was measured with the micrometer caliper (Mitutoyo Corporation, Kanagawa, Japan). The film surface morphologies were observed under a field emission scanning electron microscope (FE-SEM, JSM-6701F0N0, JEOL, Tokyo, Japan) at an accelerating voltage of 5 kV. For FE-SEM observation, a thin Pt-Pd layer (12.5 nm) was sputtered on each films.

### 2.3. Surface Wettability and Water Vapor Adsorption of Thin Films

The hydrophilicity of the film surface was evaluated from the static contact angle measurement towards distilled water (4 µL) and measured with the Pocket Goniometer PG-3 (Matsubo Corporation, Tokyo, Japan) at room temperature by the sessile-drop method (n = 3). N_2_ adsorption isotherms of the films were obtained using a surface area and pore size distribution analyzer (BELSORP MINI X, Microtrac BEL, Inc., Osaka, Japan). The specific surface area was derived using the Brunauer–Emmett–Teller (BET) N_2_ adsorption method. The films were cut into small pieces and pretreated at 150 °C for 15 h using a vacuum heating pretreatment device (BELPREP VAC II, Microtrac BEL, Inc., Osaka, Japan). The water vapor adsorption isotherms of the films were measured using a BELSORP-18 (Microtrac BEL, Inc., Osaka, Japan) at 25 °C.

Distilled water was dropped onto the prepared thin film to investigate the stability of the film morphology wet conditions. The appearances of the thin films were observed after 10 s.

### 2.4. Residual Amino Groups of Thin Films

The degree of crosslinking was evaluated using the ninhydrin assay [14] and defined as the percentage of free amino groups in the films (n = 3). Circular punched films with a diameter of 6 mm were immersed in the ninhydrin solution (Wako Pure Chemical Industries, Ltd., Osaka, Japan, 1 mL buffer solution/0.6 mL ninhydrin solution) and kept at 80 °C for 20 min. The solution was then cooled to room temperature and the optical absorbance of the supernatant solution was recorded at 570 nm with a micro-spectrophotometer (DS-11+, DeNovix, Wilmington, DE, USA). The percentage of free amino groups in the sample was determined using Equation (1).

Free amino groups (%) = 100 × (Film absorbance/Absorbance of Ch film)(1)

### 2.5. Microstructure of Thin Films

X-ray photoelectron spectroscopy (XPS) was carried out using an AXIS-NOVA (Shimadzu, Kyoto, Japan), equipped with Al Kα radiation as the monochromatic X-ray source, at 150 W. XPS spectra were collected through a hemispherical analyzer at a pass energy of 20 eV and energy step size of 1 eV with a charge-neutralizing gun. The binding energy (BE) was normalized to the C_1s_ peak (284.6 eV; 1 eV = 1.602 × 10^−9^ J) of adventitious carbon accumulated in the analysis chamber of the spectrometer and the BE values are presented in eV after convention. The spectral profiles were deconvolved with a few traces of the (Gauss (30%)–Lorentz (70%)) mixed function.

Solid-state NMR measurements were carried out using an Agilent DD2 500 MHz NMR spectrometer (Agilent Technologies, Inc., Santa Clara, CA, USA) operating at 11.7 Tesla. A zirconia rotor with a diameter of 3.2 mm was used with an Agilent HXY T3 MAS probe. The rotor spinning frequency for magic angle spinning (MAS) was controlled to be 15 kHz. ^1^H→^13^C cross-polarization (CP)-MAS NMR experiments were operated at resonance frequencies of 499.76 and 125.68 MHz for ^1^H and ^13^C, respectively, using 2.3 μs pulse length (π/2-pulse angle) for ^1^H, 500 μs contact time and 10 s recycle delays. The signals from 7400 to 8140 pulses were accumulated for each film, with adamantane (C_10_H_16_) as the external reference (38.52 ppm vs. 0 ppm TMS). ^1^H→^29^Si CP-MAS NMR experiments were performed at resonance frequencies of 499.76 and 99.28 MHz for ^1^H and ^29^Si, respectively, using 2.3 μs pulse length (π/2-pulse angle) for ^1^H, 5 ms contact time and 5 s recycle delays. The signals from 16,400 to 13,000 pulses were accumulated for each film, with polydimethylsilane (PDMS) as the external reference (−34.44 ppm vs. 0 ppm TMS).

### 2.6. Mechanical Properties of the Thin Films

**Figure 1:**
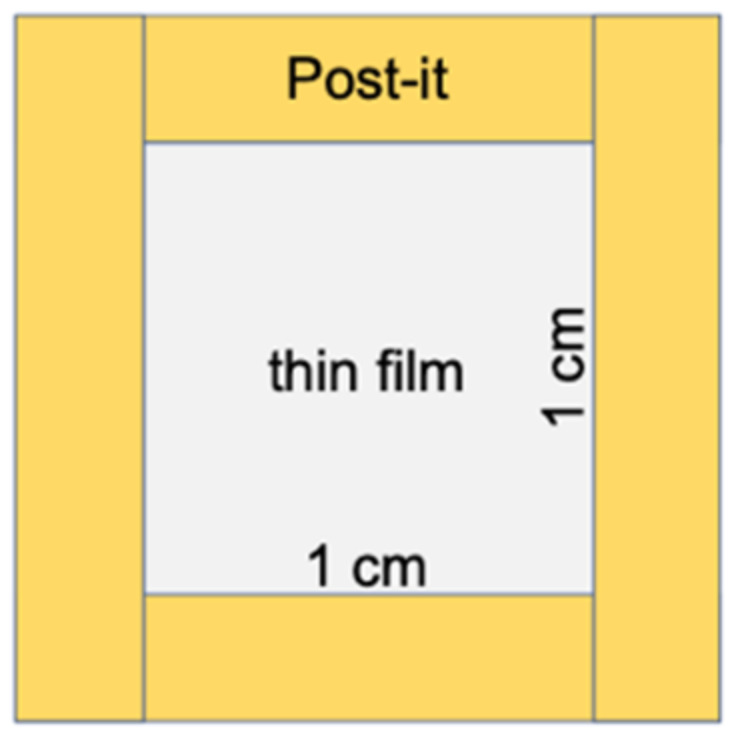
Diagram of the sample setup for the puncture test.

For the puncture test (JIS Z 1707), the film was fixed using an instant glue to a Post-it note (Figure 1). The puncture test (n = 3) was carried out with a creep meter (RE2-3305C, Yamaden, Tokyo, Japan) at a measurement speed of 1 mm·s^−1^ and contact surface diameter of 1 mm.

### 2.7. Release of Amount of BZ from the Thin Films

Punched films were soaked in phosphate buffered saline (PBS(−), pH = 7.4) and kept at 37 °C for 1 day. Then, the optical absorbance of the supernatant solutions was recorded at 250 nm with a micro-spectrophotometer (DS-11+, DeNovix, Wilmington, DE, USA). The released amount of BZ in PBS(−) were determined using a standard curve and calculated per sample. The released ration was calculated using Equation (2).

The released ratio (%) = 100 × (the released amount of BZ (µg)/the doped amount of BZ (µg)/sample(2)

### 2.8. In Vitro Antibacterial Properties Test

The antibacterial activity was tested against *Staphylococcus aureus* (*S. aureus* 209P, ATCC 6538P) using the disk diffusion method (Clinical and Laboratory Standards Institute, CLSI, M02-A12). The punched films sterilized with ethylene oxide gas were placed on the agar plates previously inoculated with *S. aureus*. The plates were incubated at 37 °C for 1 day. The zones of inhibition were measured on the basis of the average diameter of the clear zone directly using a ruler with the unaided eye from the underside of the petri dish.

### 2.9. In Vitro Cytotoxicity Test

The cytotoxicity of the films was estimated from the viability of L929 fibroblasts (KAC Co., Ltd., Kyoto, Japan). L929 fibroblasts were cultured at 37 °C in a humidified atmosphere of 5% CO_2_ in air, in an eagle’s minimum essential medium (E-MEM, MP Biomedicals, Santa Ana, CA, USA) with 10% fetal bovine serum (FBS, Gibco, Waltham, MA, USA), 100 U·mL^−1^ penicillin and streptomycin solution (Gibco, Waltham, MA, USA) and 2.5 μg·mL^−1^ fungizone (Gibco, Waltham, MA, USA). After culturing for 3 days (Cells covered 80% of plate), the sterilized film was introduced with an insert (filter pore size 8.0 μm) and cultured for 24 h at 37 °C in a humidified atmosphere of 5% CO_2_ in air. The cells with only insert is denoted control. The seeded specimens were then incubated with 5 mg·mL^−1^ 3-[4,5-dimethylthiazol-2-yl]-2,5-diphenyltetrasodium bromide (MTT, Sigma-Aldrich, St. Louis, MO, USA) at 37 °C for 4 h. The medium was decanted and then the obtained formazan crystals were dissolved with dimethyl sulfoxide (Wako Pure Chemical Industries, Ltd., Osaka, Japan) and the absorbance was measured at 560 nm with a multifunctional microplate reader (Thermo Scientific, Waltham, MA, USA). The cell viability was determined using Equation (3).

The cell viability = 100 × (Abs560 nm with films/Abs560 nm without films and insert)(3)

### 2.10. Statistical Analysis

The biological tests were carried out in triplicates and the results were presented as means ± standard derivation (SD). The statistical analysis were performed using one-way analysis of variance (ANOVA) followed by Tukey’s test with a significance level of * *p* < 0.05 or ** *p* < 0.01.

## 3. Results and Discussion

### 3.1. Surface Morphology and Hydrophilicity of the Thin Films

**Figure 2:**
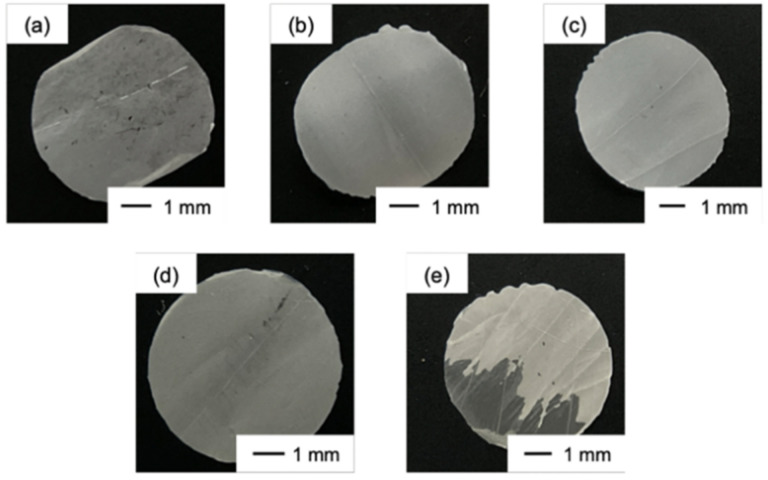
Digital images of the thin films: (**a**) Ch; (**b**) ChG05; (**c**) ChG10; (**d**) ChG05B1; (**e**) ChG05B5.

**Figure 3:**
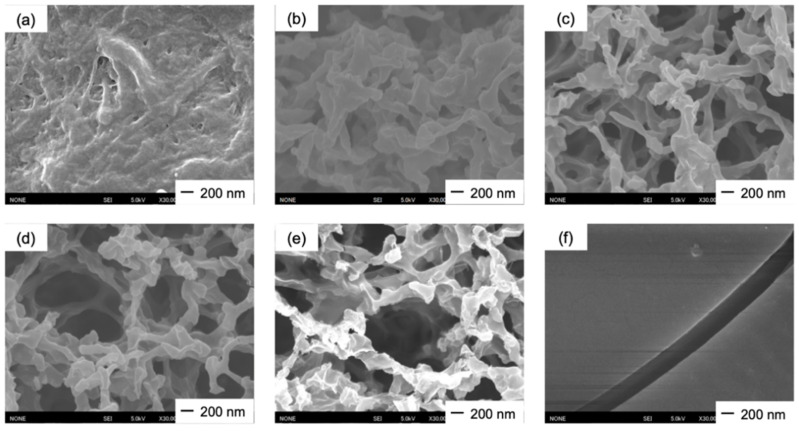
FE-SEM images of the thin film surfaces: (**a**) Ch; (**b**) ChG01; (**c**) ChG05; (**d**) ChG10; (**e**) ChG05B1; (**f**) ChG05B5.

The thicknesses of the obtained films were in the range 10–20 µm and the surface pH was approximately 6.0 in all cases. Ch had low strength and became wrinkled at the edge after being punched (Figure 2a). The ChG films (Figure 2b,c) were white and ChG05B1 and ChG05B5 (Figure 2d,e) were yellowish as a result of the addition of BZ. ChG05B5 (Figure 2e) was translucent at some areas. FE-SEM images confirmed the interconnected pores structure on the thin film surfaces (Figure 3). The Ch surfaces had a chitosan nanofiber aggregate structure with densely packed nanosized-fine fibers (Figure 3a). The addition of GPTMS led to the formation of larger pores with hundreds nanometer-scale diameter (Figure 3b,d). The size of the nanofiber bundles was approximately 100 nm and they contained 2–5 fibers. The porous structures were maintained when BZ was added (Figure 3e). However, the excess BZ covered the thin film surfaces (ChG05B5) and filled the pores (Figure 3f). The translucent appearance of ChG05B5 (Figure 2e) is attributed to the deposition of BZ on the thin film surfaces.

**Figure 4:**
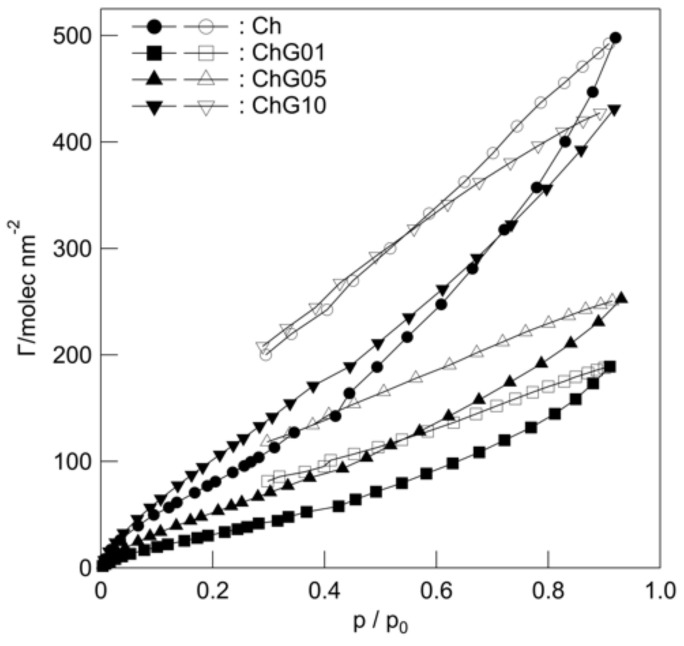
Water vapor adsorption-desorption on the thin films at 198 K (closed symbols: adsorption; open symbols: desorption).

The contact angles (Table 2) decreased in the order: Ch (91.9 ± 9.8°) > ChG01 (80.8 ± 4.9°) > ChG05 (76.4 ± 2.0°) > ChG10 (71.1 ± 3.6°), indicating that the addition of GPTMS improved the wettability of the films. The addition of BZ reduced the contact angle with water. BZ was expected to be easily released under wet conditions. The specific BET surface areas of the thin films were in the range 11.0 m^2^ g^−1^ to 47.8 m^2^ g^−1^ (Table 3). The water vapor adsorption and desorption isotherms of the films (Figure 4) at 298 K exhibited type II shape, according to the standard classification of the International Union of Pure and Applied Chemistry (IUPAC) [22]. The water vapor adsorption and desorption curves did not coincide with the tendency of wettability and all films showed hysteresis behavior at the high relative pressure. This type II shape suggests that the thin films had pores that were greater than 50 nm in diameter and that the pores filled with the multilayers of adsorbed water at high relative pressure. The desorption curve was above the adsorption curve and the hysteresis loop did not closed (low pressure hysteresis) [23]. This indicates that the desorption of water was more difficult than adsorption owing to the capillary forces. The amount of water adsorption was lower for ChG01 and ChG05 than for Ch and ChG10.

### 3.2. Microstructure and Mechanical Properties of the Thin Films

**Figure 5:**
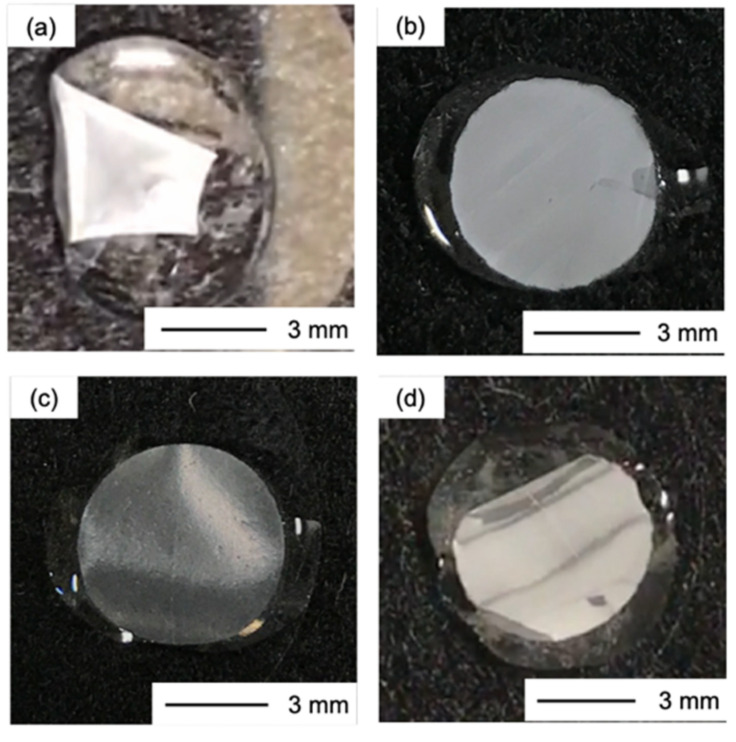
The digital images after dropping a few drops of water: (**a**) Ch; (**b**) ChG01; (**c**) ChG05; (**d**) ChG05B1.

**Figure 6:**
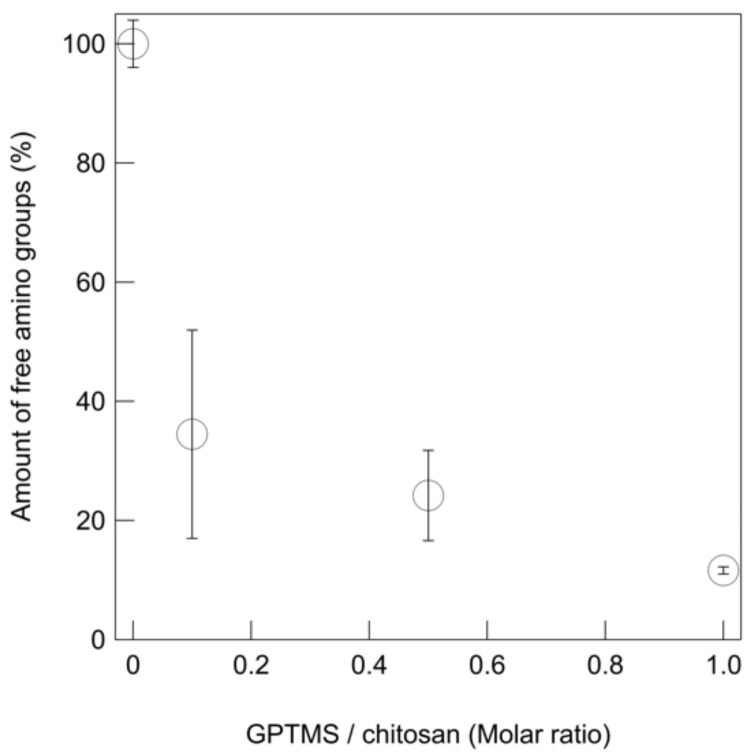
Residual amino groups of the thin films with different amount of GPTMS measured by ninhydrn reaction.

Dropping water onto the thin films caused Ch to immediately shrink, while the ChG series showed no change in appearance, even when the amount of GPTMS added was small (Figure 5 and Video S1 and Video S2). The addition of BZ led to a slight recovery of the instability toward water. The amount of free amino groups in the films was estimated using the ninhydrin method (Figure 6). The number of free amino groups was not proportional to the amount of GPTMS. The results suggest that the percentage of free amino groups was 30% in ChG01 (chitosan/GPTMS = 0.1 molar ratio), 20% in ChG05 (chitosan/GPTMS = 0.5 molar ratio) and 10% in ChG10 (chitosan/GPTMS = 1.0 molar ratio). The presence of positively charged amino groups and negatively –Si–O^−^ groups favors electrostatic interactions [14,15,16,17]. Therefore, in ChG01 and ChG05 chitosan and GPTMS interacted through ring-opening reactions and electrostatic interactions. In case of ChG01, 10% is from the ring-opening and 60% is from the ionic interaction. In ChG01, polycondensation between Si–OH groups is less likely than for the other films because of the small amount of GPTMS and the remaining Si–OH groups can interact with the amino groups. As the amount of GPTMS added increased, the polycondensation of Si–OH groups led to the formation of Si–O–Si networks; therefore, the electrostatic interaction decreased, but the degree of epoxy ring-opening increased.

**Figure 7:**
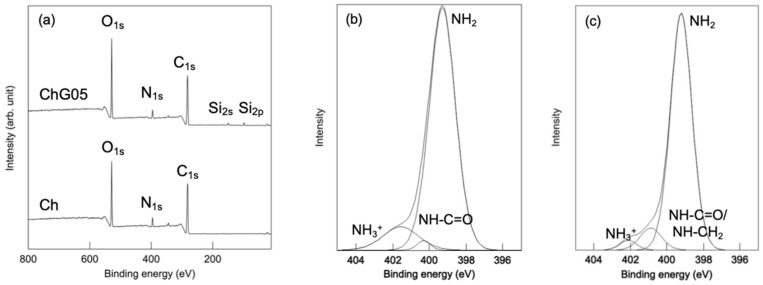
The XPS spectra of the thin films. (**a**) Wide Scan and N_1s_ spectra of (**b**) Ch and (**c**) ChG05.

The wide scan XPS spectra of both Ch and ChG05 (Figure 7, Table 4) detected O_1s_, N_1s_ and C_1s_ peaks at around 540.0, 400.0 and 290.0 eV, respectively. Peaks at 150.0 and 99.0 eV assigned to Si_2s_ and Si_2p_ [24], respectively, were only detected for ChG05. The presence of nitrogen in three different chemical states on the thin films surfaces was confirmed from their N_1s_ XPS spectra (Figure 7b,c). Ch had a strong N_1s_ peak at 399.3 eV attributed to the nitrogen atom in the –NH_2_ group and two less intense peaks at 400.3 and 401.6 eV, which suggest the presence of nitrogen atoms from the acetyl amine, –NH–C = O and protonated amino group, –NH_3_^+^ [25]. For ChG05, the relative peak area of the –NH_3_^+^ peak decreased and that of the –NH–C = O peak increased, which indicates that –NH–CH_2_ formed as a result of the reaction between protonated amino groups and epoxy groups of GPTMS. The small changes in the N_1s_ spectra were consistent with the results of the ninhydrin experiment.

**Figure 8:**
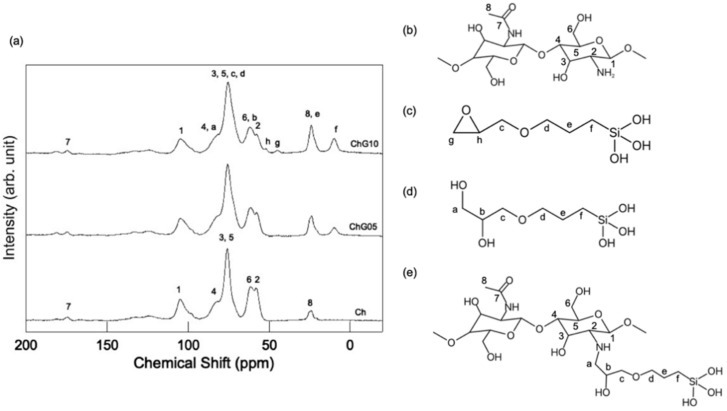
^13^C CP-MAS NMR spectra of the thin films (**a**) and the molecular structures of (**b**) chitosan, (**c**) hydrolyzed GPTMS, (**d**) ring-opened and hydrolyzed GPTMS and (**e**) chitosan-GPTMS.

**Figure 9:**
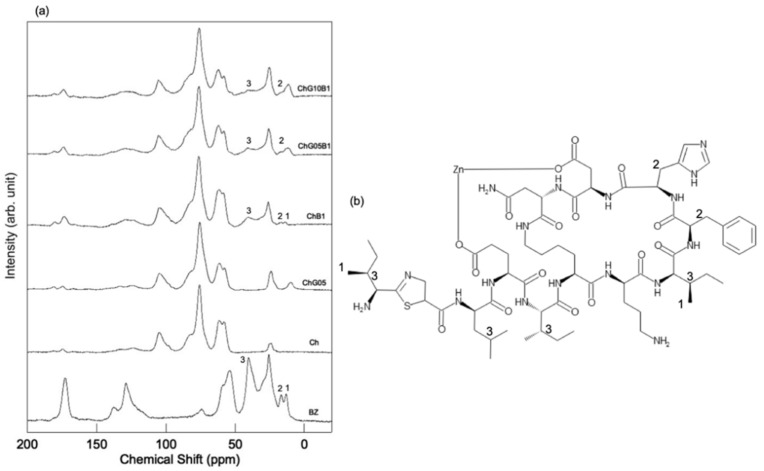
^13^C CP-MAS NMR spectra of the thin films with BZ (**a**) and the molecular structure of BZ (**b**).

Chitosan matrix signals appeared at 105.1 (C1), 82.6 (C4), 75.8 (C3 and C5), 61.6 (C6), 58.1 (C2), 174.4 (N-acetyl C = O signal) and 24.1 (N-acetyl-methyl CH_3_ signal) ppm in the ^13^C CP-MAS NMR spectra (Figure 8 and Figure 9) [26,27,28]. The intensity of C2/C1 in the Ch, ChG05 and ChG10 spectra was 1.52, 1.29 and 1.28. The lower C2/C1 for ChG05 and ChG10 compared with Ch suggested that the amino groups of chitosan reacted with GPTMS. The Cg and Ch peaks corresponding to non ring-opened epoxy groups were detected for ChG10. These peaks are attributed to GPTMS that did not react with the amino groups of chitosan, which correspond to the residual amino groups determined using the ninhydrin methods. The spectra also confirmed the addition of BZ to the films (Figure 9a). The main peaks of the thin films did not change and the carbons of the methyl groups at 13.0 ppm, the CH_2_ carbons adjacent to the cyclic structures at 16.5 ppm and the tertiary carbons with methyl and ethyl groups at 40.3 ppm, were detected and corresponded to BZ [29]. These observations indicate that BZ did not react with the thin film matrix.

**Figure 10:**
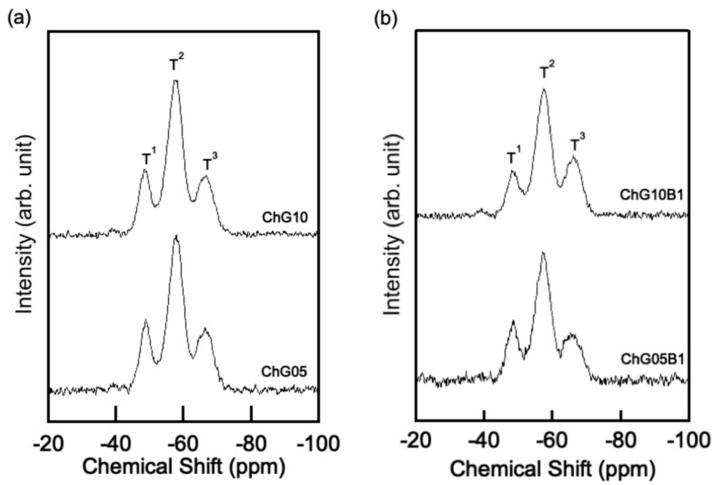
^29^Si CP-MAS NMR spectra of the thin films, (**a**) without BZ and (**b**) with BZ.

The ^29^Si CP-MAS NMR spectra (Figure 10) demonstrate the change of the methoxysilane group of GPTMS due to hybridization. The profiles were deconvolved into three component peaks due to T^1^, T^2^ and T^3^ siloxane units, from which the chemical shift, full-width at half-maximum and relative peak area (%) of each T^n^ unit were calculated as in a previous study [14,15,16] (Table 5). T^1^, T^2^ and T^3^ denote R–Si(–OSi)(OCH_3_, OH)_2_, R–Si(–OSi)_2_(OCH_3_, OH) and R–Si(–OSi)_3_, respectively (R is the organic skeleton derived from GPTMS). The number of Si–O–Si bridging bonds per Si atom, N_bo_/Si, derived from condensation of the –Si–OH or –Si–OCH_3_ groups at the silane end of a GPTMS molecule, is given by Equation (4), according to simple valence theory:

N_bo_/Si = (fraction of T^1^) × 1 + (fraction of T^2^) × 2 + (fraction of T^3^) × 3 (4)

The N_bo_/Si in each sample was approximately 2.0 regardless of the amount of GPTMS and BZ. The addition of BZ did not change the structure surrounding S, which indicates that BZ interacted with the thin film matrix by intermolecular forces without forming a chemical bond.

**Figure 11:**
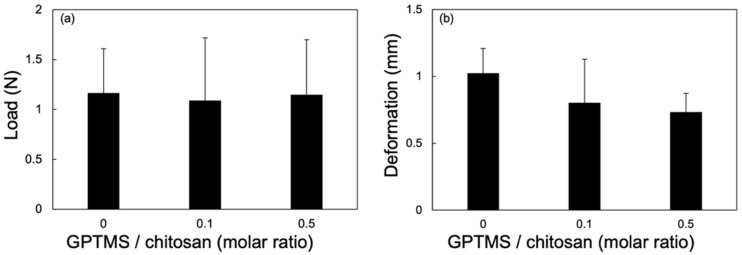
Mechanical properties determined using a puncture strength test: (**a**) breaking load; (**b**) deformation.

The mechanical properties of the films were examined using a puncture strength test (Figure 11). ChG10 was too brittle for the puncture strength test to be carried out. The maximum breaking load was the same with and without GPTMS; however, Ch had greater deformation than ChG01 and ChG05.

**Figure 12:**
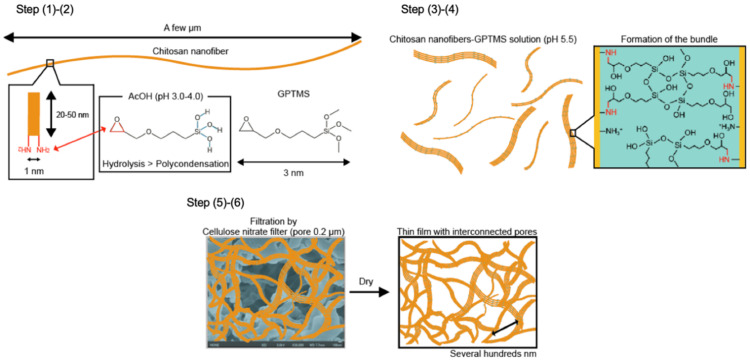
Illustration of the interconnected pore formation.

Chitosan nanofibers are a few μm length and the distance between amino groups of chitosan is about 1 nm [30]. The length of GPTMS molecule is about 3 nm [31] and should be branched on the chitosan nanofiber surface by open-ring reaction. GPTMS are also hydrolyzed in acetic acid (pH 3.0–4.0) to form –Si–OH groups because the hydrolysis of methoxy groups preferentially occur, rather than polycondensation of –Si–OH groups at pH lower 5.5 [32]. Based on the structure analysis, the interconnected porous structure formation is thought to follow the following steps (Figure 12): (1) The chitosan nanofibers are dispersed in aqueous solution; (2) GPTMS hydrolyzed by acetic acid to form –Si–OH groups and react with the chitosan nanofibers through opening ring reaction of epoxide; (3) the 2–5 nanofibers aggregated and formed the bundles by polycondensation of GPTMS; (4) The –Si–OH groups are also interacted with the remained amino groups; (5) the bundles form a wall and separate from the water phase on the cellulose nitrate filter; (6) finally, the water is evaporated and the interconnected pores are formed with the bundles forming the walls. The polycondensation of GPTMS are also promoted under dry. The –Si–OH groups and –Si–O–Si– networks improved the surface wettability and reduced the flexibility. The water adsorption depended on the remaining amino groups and the amount of –Si–OH groups and –Si–O–Si– networks from GPTMS.

### 3.3. Release of BZ and In Vitro Biological Properties

**Figure 13:**
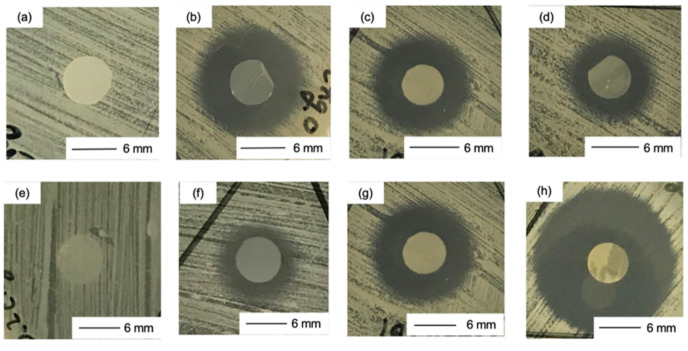
Digital images of the disc diffusion test against *S. aureus*. (**a**) Ch; (**b**) ChB1; (**c**) ChG05B1; (**d**) ChG10B1; (**e**) ChG05; (**f**) ChG05B05; (**g**) ChG05B1; (**h**) ChG05B5.

The amount of BZ released from the films after 1 day was small, even in PBS. The release on the agar medium was expected to be slower. As the amount of GPTMS increased, the release of BZ was suppressed (Table 6). In addition, as the amount of BZ loaded into the films increased, the amount released also increased. The bacitracin molecules interacted with the thin film matrix through the –Si–OH groups derived from GPTMS and the release was suppressed as the amount of GPTMS increased. However, the amount of BZ that could be loaded into the matrix was limited and it easily diffused out at high concentrations of BZ. Ch and ChG05 did not show antibacterial properties against *S. aureus* (Figure 13 and Table 6). This indicates that the amino groups on these films were not protonated under biological conditions. The antibacterial effect depended on the amout of BZ released. ChG05B5 showed a larger inhibition zone (* *p* < 0.05), 21.3 ± 0.1 mm than ChG05B1, 14.1 ± 0.6 mm. The addition of GPTMS slightly suppressed (** *p* < 0.01) the inhibition of *S. aureus*, but the difference was not marked. The Zn^2+^ of BZ electrostatically attached to the negatively charged bacterial surface, where BZ was able to prevent cell wall and enzyme synthesis [21]. Amino groups in the thin films were able to form complexes with Zn^2+^, which retarded the release of BZ.

**Figure 14:**
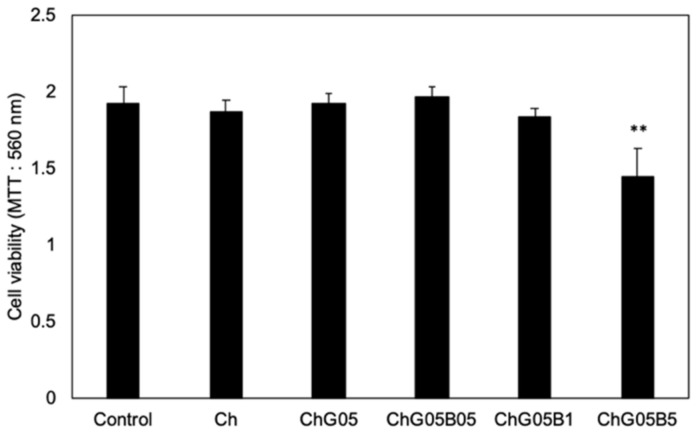
The cell viability of L929 fibroblasts after incubation with the thin films for 24 h (** *p* < 0.01).

**Figure 15:**
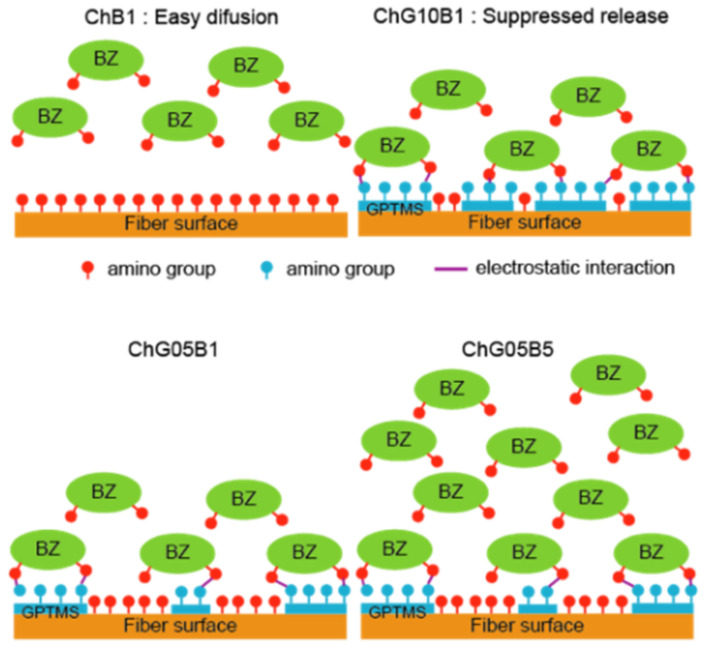
Illustration of the release of BZ from the thin films.

The effect of BZ on L929 fibroblasts was demonstrated by measuring the cell viability when cultured with an insert (Figure 14). The films did not touch the cell surface and we were able to examine the effect of the released BZ without shutoff between the cells and medium. Ch, ChG05, ChG05B05 and ChG05B1 did not show toxicity towards L929 and samples exhibited the same cell viability as the Control (medium only). Only cells expose to ChG05B5 showed approximately 25% lower viability than those exposed to the other films and the Control. We concluded the release of BZ between ChB1 and ChG10B1 and ChG05B1 and ChG05B5 in Figure 15. ChB1 does not have the electrostatic interaction with BZ and released easily. On the other hand, ChG10B1 surface has –Si–OH groups and interact with amino groups of BZ to suppress the release. The higher released of BZ from ChGB1 showed higher antibacterial effects on *S. aureus* than ChG10B1. Between ChG05B1 and ChG05B5, the amount of the interacted BZ on the chitosan fiber surfaces are same and the release depends on the starting amounts to occur the excess release. This excess release inhibited the viability of fibroblast. Zn^2+^ ions easily absorbed on the cell surfaces and then bacitracin covered them to interfere with supply of nutrients to the cells.

## 4. Conclusions

Chitosan nanofibers-based thin films with interconnected pores were prepared by crosslinking with GPTMS. Epoxy groups and silanol groups from GPTMS reacted with amino groups chitosan nanofibers and improved the stability in water and surface wettability of the thin films. Silanol groups also formed siloxane network by the polycondensation. Bacitracin zinc salt was dosed into the films through the electrostatic interactions. An appropriate amount of BZ release from the thin film was achieved that showed antibacterial effects against *S. aureus* without exhibiting toxicity towards fibroblasts. Chitosan nanofibers-based thin films with BZ show potential for use in the prevention of skin infections even at neutral pH condition.

## Figures and Tables

**Table 1:** Initial molar ratio of GPTMS to the amino groups of chitosan and BZ concentration in the starting solutions.

Sample	GPTMS/Chitosan Amino Groups (Molar Ratio)	BZ Concentration (g L^−1^)
Ch	1:0	0
ChG01	1:0.1	0
ChG025	1:0.25	0
ChG05	1:0.5	0
ChG05B05	1:0.5	0.77
ChB1	1:0	1.54
ChG05B1	1:0.5	1.54
ChG10B1	1:1	1.54
ChG05B5	1:0.5	7.7

**Table 2:** Static contact angle (°) toward distilled water for the thin films surface.

Ch	ChG01	ChG05	ChG10	ChB1	ChG05B05	ChG05B1	ChG05B5	ChG10B1
91.9 ± 9.8	80.8 ± 4.9	76.4 ± 2.0	71.1 ± 3.6	70.0 ± 10.6	66.1 ± 0.7	52.5 ± 4.3	42.6 ± 5.0	55.7 ± 5.1

**Table 3:** Specific BET surface area of the thin films.

Sample	SSA (m^2^ g^−1^)
Ch	17.8
ChG01	47.8
ChG05	28.1
ChG10	11.0

**Table 4:** N_1s_ binding energy (BE (eV)), full width at half maximum (FWHM (eV)) and relative area (I (%)) from the XPS spectra.

Sample	–NH_3_^+^			–NH–CH_2_–/–NH–C = O			–NH_2_		
	BE ^a^	FWHM ^b^	I ^c^	BE ^a^	FWHM ^b^	I ^c^	BE ^a^	FWHM ^b^	I ^c^
Ch	401.570	2.330	11.5	400.314	0.848	1.7	399.292	1.695	86.8
ChG05	402.115	1.102	2.9	400.889	1.398	8.3	399.218	1.399	88.8

^a^ Estimated error of binding energy < ±0.001 eV. ^b^ Estimated error of full width half maximum < ±0.003 eV. ^c^ Estimated error of relative area < ±0.1%.

**Table 5:** ^29^Si chemical shift (δ (ppm)), full width at half maximum (FWHM (ppm)) and relative area (I (%)) for T unit derived from the ^29^Si CP-MAS NMR spectra.

Sample	T^1^			T^2^			T^3^			N _bo_/Si
	δ ^a^	FWHM ^b^	I ^c^	δ ^a^	FWHM ^b^	I ^c^	δ ^a^	FWHM ^b^	I ^c^	
ChG10	−49.02	4.69	19.69	−57.87	5.48	57.15	−66.76	5.80	23.16	1.96
ChG05	−48.92	4.48	19.90	−57.75	5.35	56.26	−66.40	5.64	23.84	1.96
ChG10B1	−48.72	4.90	17.08	−57.40	5.25	55.19	−66.10	5.68	27.73	2.11
ChG05B1	−48.54	4.74	21.18	−57.25	5.43	55.38	−65.84	6.07	23.44	2.02

^a^ Estimated error of chemical shifts < ±0.03 ppm. ^b^ Estimated error of full width half maximum < ±0.06 ppm. ^c^ Estimated error of relative area < ±0.28%.

**Table 6:** BZ release after 1-day immersion in PBS(−) and antibacterial effect against *S. aureus*. (* *p* < 0.05, ** *p* < 0.01).

Sample	BZ Release (µg/Sample) and Ratio (%)	Inhibition Zone (mm)
Ch	0	0
ChB1	13.0 (3.0%)	17.5 ± 2.3
ChG05	0	0
ChG05B05	3.2 (1.5%)	14.9 ± 4.8
ChG05B1	11.9 (2.8%)	14.1 ± 0.6
ChG05B5	49.4 (2.3%)	21.3 ± 0.1 *
ChG10B1	8.4 (1.9%)	11.5 ± 0.3 **

## Data Availability

The data that support the findings of this study are available from the corresponding author, upon reasonable request.

## Supplementary Materials

The following are available online at https://www.mdpi.com/article/10.3390/polym13071104/s1, Video S1: Shape changes of Ch after dropping water on the surface and Video S2: Shape changes of ChG05 after dropping water on the surface.
